# Local icariin application enhanced periodontal tissue regeneration and relieved local inflammation in a minipig model of periodontitis

**DOI:** 10.1038/s41368-018-0020-3

**Published:** 2018-06-12

**Authors:** Xiuli Zhang, Nannan Han, Guoqing Li, Haoqing Yang, Yangyang Cao, Zhipeng Fan, Fengqiu Zhang

**Affiliations:** 1 Department of Periodontology, Capital Medical University School of Stomatology, Beijing, China; 2 Laboratory of Molecular Signaling and Stem Cells Therapy, Beijing Key Laboratory of Tooth Regeneration and Function Reconstruction, Capital Medical University School of Stomatology, Beijing, China; 3 Molecular Laboratory for Gene Therapy and Tooth Regeneration, Beijing Key Laboratory of Tooth Regeneration and Function Reconstruction, Capital Medical University School of Stomatology, Beijing, China

## Abstract

Periodontitis is an inflammatory autoimmune disease. Treatment should alleviate inflammation, regulate the immune reaction and promote periodontal tissue regeneration. Icariin is the main active ingredient of Epimedii Folium, and it is a promising compound for the enhancement of mesenchymal stem cell function, promotion of bone formation, inhibition of bone resorption, alleviation of inflammation and regulation of immunity. The study investigated the effect of icariin on periodontal tissue regeneration in a minipig model of periodontitis. The minipig model of periodontitis was established. Icariin was injected locally. The periodontal clinical assessment index, a computed tomography (CT) scan, histopathology and enzyme-linked immune sorbent assay (ELISA) were used to evaluate the effects of icariin. Quantitative analysis results 12 weeks post-injection demonstrated that probing depth, gingival recession, attachment loss and alveolar bone regeneration values were (3.72 ± 1.18) mm vs. (6.56 ± 1.47) mm, (1.67 ± 0.59) mm vs. (2.38 ± 0.61) mm, (5.56 ± 1.29) mm vs. (8.61 ± 1.72) mm, and (25.65 ± 5.13) mm^3^ vs. (9.48 ± 1.78) mm^3^ in the icariin group and 0.9% NaCl group, respectively. The clinical assessment, CT scan, and histopathology results demonstrated significant enhancement of periodontal tissue regeneration in the icariin group compared to the 0.9% NaCl group. The ELISA results suggested that the concentration of interleukin-1 beta (IL-1β) in the icariin group was downregulated compared to the 0.9% NaCl group, which indicates that local injection of icariin relieved local inflammation in a minipig model of periodontitis. Local injection of icariin promoted periodontal tissue regeneration and exerted anti-inflammatory and immunomodulatory function. These results support the application of icariin for the clinical treatment of periodontitis.

## Treating periodontitis with Chinese herbal injection

A Chinese herbal supplement could treat an inflammatory autoimmune disease of the tissues surrounding the teeth. Zhipeng Fan and colleagues at Capital Medical University in Beijing, China found reduced inflammation and improved tissue regeneration in induced periodontitis in miniature pigs, three months following local injections with the Chinese herbal extract icariin. Periodontitis is an overactive immune response to bacterial invasion in the gums, leading to the breakdown of supportive tissue and bone, and potential tooth loss. Clinical assessment, CT scans and microscopic examination showed icariin injections promoted gum and bone regeneration, possibly by stimulating local stem cells. It also reduced signs of inflammation, including significantly decreasing the expression of IL-1β, an immune response mediator. Further studies are needed to confirm icariin’s mode of action and optimal dosage for potential clinical use.

## Introduction

Periodontitis is a common chronic inflammatory autoimmune disease that is characterised by the loss of periodontal support tissues, which is an important cause of tooth loss in adults. Periodontopathic bacteria initiate periodontitis, but the overactive response of the host immune system against bacterial invasion likely plays an essential role in the direct or indirect modulation of osteoblast and osteoclast formation, which leads to the breakdown of connective tissue attachment and alveolar bone.^1,2^ There is an urgent need to develop a treatment method to alleviate inflammation, regulate the immune reaction, and balance the osteogenesis and osteoclasis to ultimately result in the enhanced regeneration of periodontal tissues. Conventional periodontitis treatment methods, such as scaling, root planning, guided tissue regeneration, and pharmacotherapy, do not achieve ideal periodontal tissue regeneration, inflammation control or immunomodulatory effects.^3^ Mesenchymal stem cell (MSC)-mediated periodontal tissue regeneration is considered an alternative method for periodontitis treatment.^4^ MSC transplantation for periodontal tissue regeneration has made remarkable strides, but several key problems for its use exist, such as security and ethics.^5^ There are other alternatives for periodontal tissue regeneration, such as chemical agents, for the treatment of periodontitis. Therefore, there is an urgent need to develop alternative drugs with lower cost and higher efficacy to alleviate inflammation, regulate immunity, and enhance the endogenous functions of MSCs to promote the regeneration of periodontal tissues.

Herbal medicines are natural products that have played important roles in disease prevention and treatment since ancient times, especially in China. The study of the therapeutic potential of herbal medicines made enormous progress in recent years. Several medicinal plants contain natural flavonoid compounds, such as Quercitrin, Black Mulberry and Epimedium species, which are abundant and ordinary but produce an array of benefits, such as antioxidant, antimicrobial, anti-apoptotic and anti-inflammatory properties.^6–8^ Several studies reported the extensive therapeutic capacities of Epimedium, such as osteoprotective effects, reproductive protective effects, neuroprotective effects, cardiovascular protective effects, anticancer effects, and immunoprotective effects.^9^ These reports described the effectiveness of Epimedium in the treatment of diseases of the skeletal system, such as osteoporosis, which strengthen tendon and bone.^10^ Icariin (ICA) (C_33_H_40_O_15_, molecular weight: 676.67) is a Chinese herbal monomer that is extracted as the main active ingredient of Epimedium. Several studies demonstrated that icariin accelerated osteoblastic differentiation, promoted bone formation and inhibited osteoclastic differentiation and bone resorption.^11,12^ Icariin stimulated the proliferation and osteogenic differentiation of human periodontal ligament stem cells (hPDLSCs) in a dose-dependent manner in a suitable concentration range from 0.001 to 1 μg · mL^−1^, but cytotoxicity limited its use at doses greater than 10 μg · mL^−1^.^13^ The researchers also found that the concentration of icariin (0.01–1 μg · mL^−1^) enhanced the impaired proliferation and osteogenic differentiation potentials of hPDLSCs caused by extracts of Porphyromonas gingivalis, and the most effective concentration of icariin was 0.1 μg · mL^−1^. Icariin was used in mouse calvarial defect models and senescence models, and the results demonstrated that icariin increased trabecular bone mineral density and improved bone mass via the promotion of bone formation.^14^ Icariin significantly reduced the inflammatory responses and alleviated the pathological changes in a mouse calvarial osteolysis model.^15^ The anti-inflammatory and immunoprotective effects of icariin were also observed in immune dysfunctional mice.^16^ Taken together, icariin, as the main active ingredient of epimedium, is a promising compound for the enhancement of MSC proliferation and differentiation, promotion of bone formation, inhibition of bone resorption, alleviation of inflammation and regulation of the immune reaction, which supports its use as a candidate agent for periodontitis treatment. However, the function of icariin in periodontitis treatment is not thoroughly elucidated.

The present study established a minipig model of periodontitis and applied icariin via local injection. Local icariin injection promoted periodontal tissue regeneration and exerted anti-inflammatory and immunomodulatory functions. These discoveries support a potential application method for periodontitis treatment in the clinic.

## Results

### Local injection of icariin improved tissue and bone regeneration in periodontal defects in a minipig model of periodontitis

**Fig. 1:**
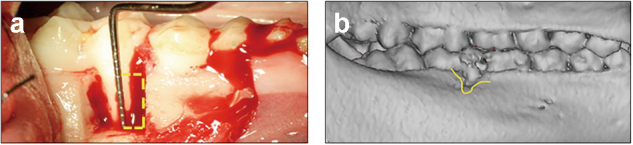
Establishment and evaluation of a minipig model of periodontitis. Generation of a minipig model of periodontitis. **a** We surgically created periodontal defects of 7 mm×5 mm×7 mm in the mesial region of mandibular first molars. **b** CT scans reveal obvious alveolar bone defects in the experimental region four weeks after the operation

**Fig. 2:**
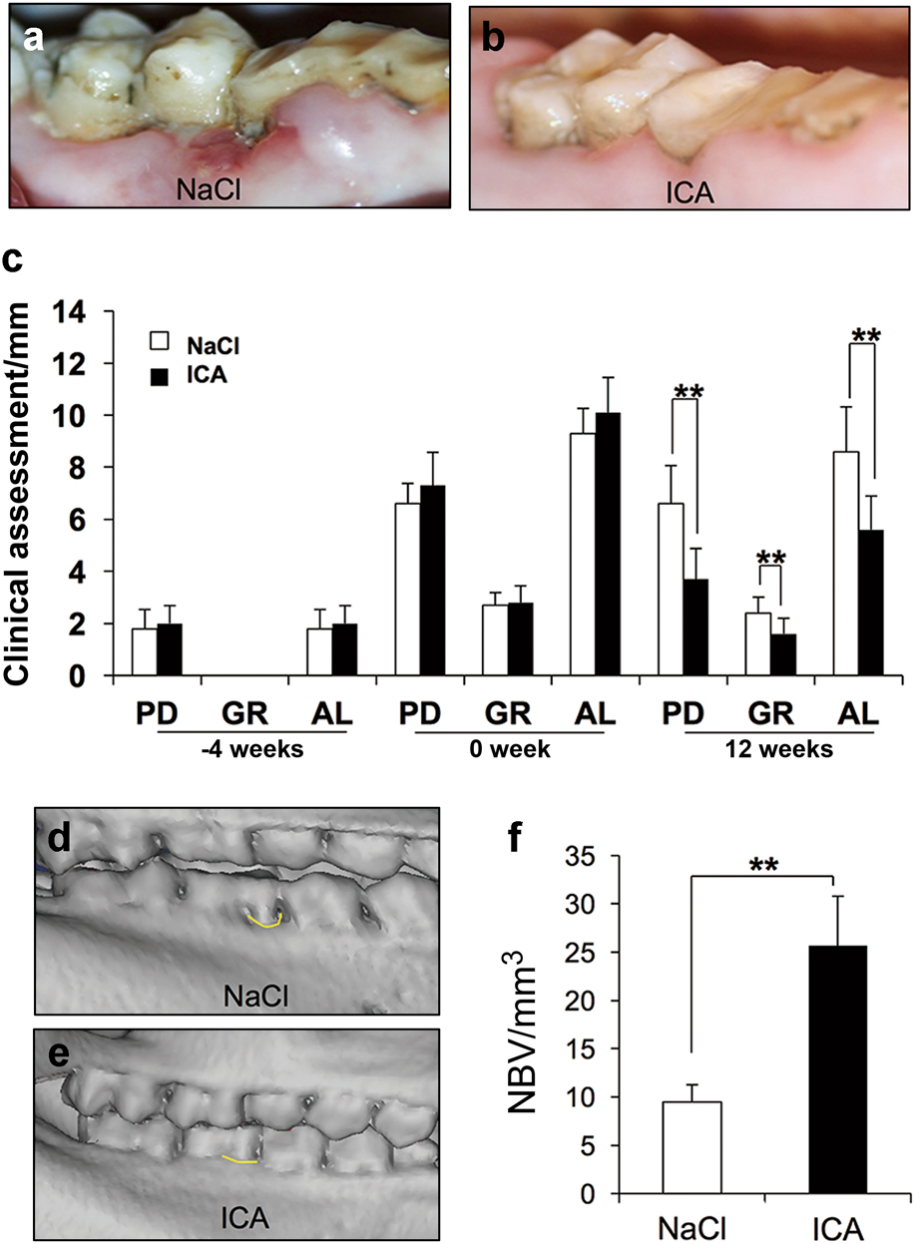
Clinical assessments and CT scans of bone tissue regeneration in a minipig model of periodontitis. **a**, **b** Intra-oral manifestations showing the general appearance of periodontal tissue in the 0.9% NaCl group and icariin group 12 weeks after injection. **c** Clinical assessment results show significantly lower PD, GR and AL values in the icariin group than in the 0.9% NaCl group 12 weeks after injection. **d**, **e** Three-dimensional reconstructive CT scans show bone formation in the 0.9% NaCl group and icariin group 12 weeks after injection. **f** Quantitative analyses of new bone formation in the 0.9% NaCl group and icariin group. Bars and vertical lines: means ± standard deviations. Student’s *t* test was used to detect statistical significance. **P < 0.01. ICA icariin; PD probing depth; AL attachment loss; GR gingival recession; NBV new bone volume

We surgically created periodontal defects (Fig. 1a) and applied a silk suture to the ligament around the cervical region of the first molars. CT examinations 4 weeks later confirmed that the minipig model of periodontitis was established (Fig. 1b). Icariin and 0.9% NaCl were injected into the periodontitis lesions. Intra-oral photographs 12 weeks after injection demonstrated distinct inflammation of periodontal tissues (red and swollen gums and obvious gingival recession (GR)) in the 0.9% NaCl group (Fig. 2a). The icariin group revealed no obvious red gums in periodontitis lesions and recovery of the gingival margin to an approximately normal level (Fig. 2b).

The general status of periodontal tissues was detected using clinical assessments. Attachment loss (AL) increased significantly from 2 to 10 mm 4 weeks after surgery, which also confirmed the successful generation of the periodontitis model. Probing depth (PD) values were (3.72 ± 1.18) mm in the icariin group and (6.56 ± 1.47) mm in the 0.9% NaCl group 12 weeks after surgery. GR values were (1.67 ± 0.59) mm in the icariin group and (2.38 ± 0.61) mm in the 0.9% NaCl group. AL values were (5.56 ± 1.29) mm in the icariin group and (8.61 ± 1.72) mm in the 0.9% NaCl group (Fig. 2c). These results indicated that local injection of icariin significantly promoted the recovery of periodontal tissues compared to the 0.9% NaCl group in a minipig model of periodontitis.

CT scans demonstrated scarce alveolar bone formation in the 0.9% NaCl group 12 weeks after injection (Fig. 2d). In contrast, local injection of icariin significantly improved the height of periodontal alveolar bone compared to 0.9% NaCl application, and it almost recovered to normal levels (Fig. 2e). Quantitative analysis results demonstrated that alveolar bone regeneration was (25.65 ± 5.13) mm^3^ in the icariin group and (9.48 ± 1.78) mm^3^ in the 0.9% NaCl group, which further confirmed that local injection of icariin markedly enhanced bone regeneration (Fig. 2f).

**Fig. 3:**
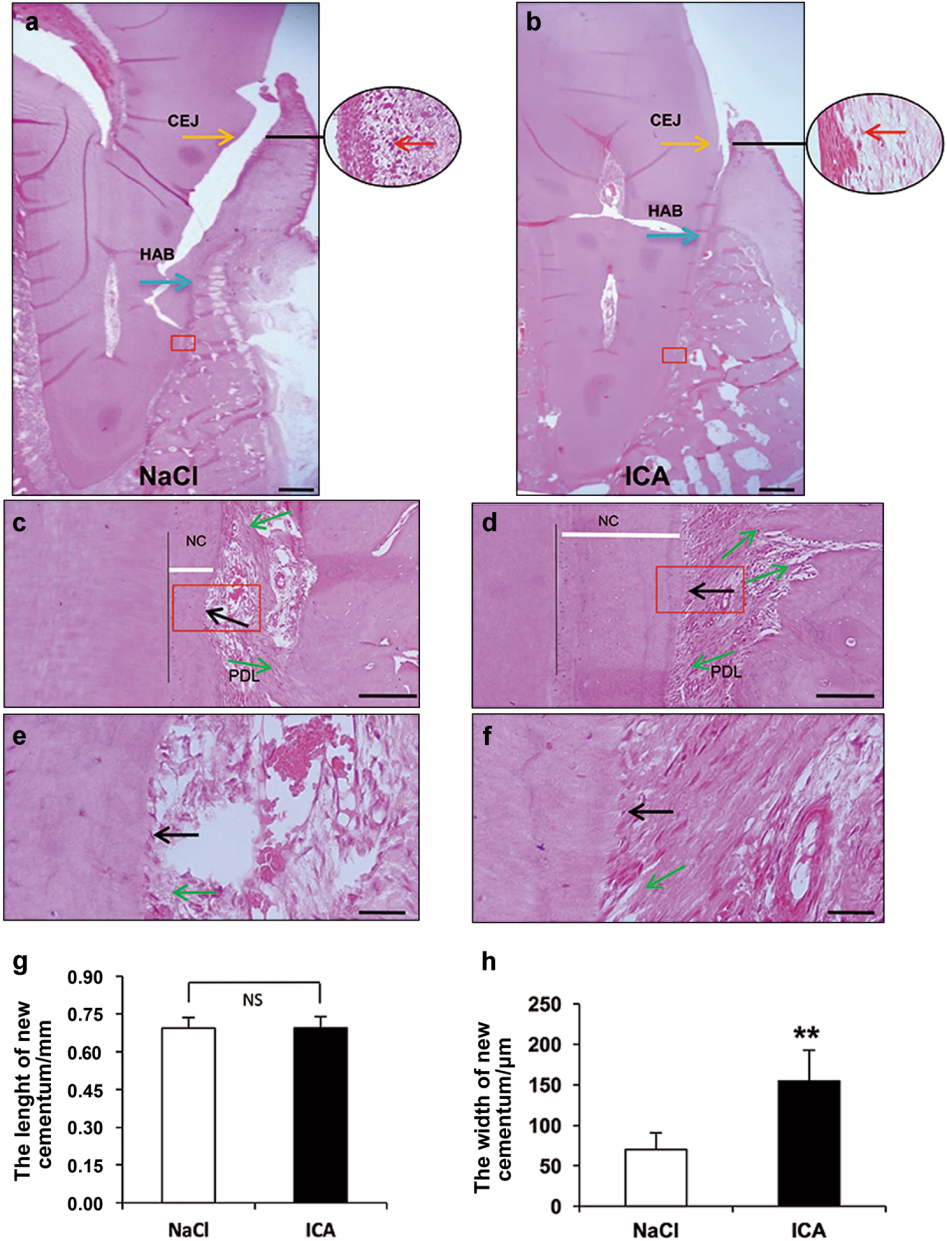
Histopathological assessments of periodontal tissue regeneration. HE staining shows new periodontal tissue regeneration in the periodontal lesions in the 0.9% NaCl group (**a**, **c**, **e**) and icariin group (**b**, **d**, **f**). HE staining reveals new cementum and Sharpey’s fibres in the 0.9% NaCl group (**c**, **e**) and icariin group (**d**, **f**). **g**, **h** Quantitative analyses of new cementum. Scale bar = 2 mm (**a**, **b**), 100 μm (**c**, **d**), 25 μm (**e**, **f**). Red arrow, inflammatory cells; Green arrow, Sharpey’s fibres; Black arrow, cementoblast; White bar = the width of new cementum. Bars and vertical lines: means ± standard deviations. Student’s *t* test was used to detect statistical significance. **P < 0.01. ICA, icariin; CEJ, cementoenamel junction; HAB, height of the alveolar bone; NC, new cementum; PDL, periodontal ligament

Periodontal tissue regeneration was also assessed using histopathological examination. H&E staining revealed that the typical periodontitis characteristics were observed in the 0.9% NaCl group, including a deep periodontal pocket, abundant inflammatory cell infiltration, and the absence of the periodontal ligament and the typical structure of Sharpey’s fibres (Fig. 3a, c, e). In contrast, a larger amount of periodontal ligament and typical structure of Sharpey’s fibres were regenerated in the icariin group (Fig. 3b). Fewer inflammatory cells and newly formed Sharpey’s fibres, which anchored into the newly regenerated cementum, were also observed in the periodontal tissues of the icariin group (Fig. 3b, d). More mature and thicker new cementum formed and more cementoblasts existed in the icariin group (Fig. 3d, f) compared to the 0.9% NaCl group (Fig. 3c, e). Quantitative analysis revealed that the length of the new cementum was not different in the icariin group and 0.9% NaCl group 12 weeks post-injection (Fig. 3g). However, the width of new cementum in the icariin group was significantly wider than in the 0.9% NaCl group (Fig. 3h).

### Local injection of icariin relieved local inflammation in a minipig model of periodontitis

**Fig. 4:**
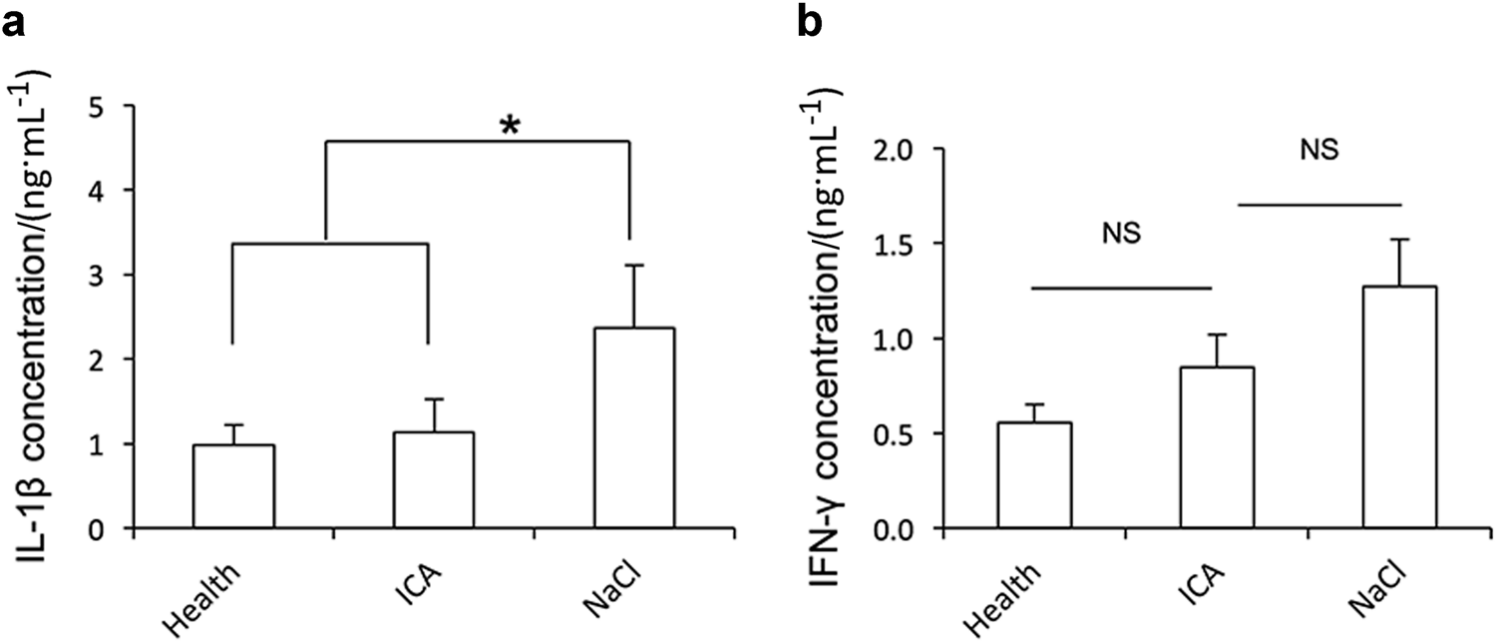
Local injection of icariin inhibits IL-1β expression in a minipig model of periodontitis. ELISA shows IL-1β **a** and IFN-γ **b** expression 12 weeks after injection. The results are presented as the means ± s.d. from three independent experiments. One-way analysis of variance was used to detect statistical significance. *P < 0.05. ICA, icariin; NS, no significant difference

The enzyme-linked immune sorbent assay (ELISA) was used to examine the inflammatory factors in gingival crevicular fluid (GCF) from minipigs. The ELISA results showed that the concentrations of interleukin-1 beta (IL-1β) and interferon-gamma (IFN-γ) in the icariin group were lower than in the 0.9% NaCl group 12 weeks after injection (Fig. 4a, b). The concentration of IL-1β was (1.14 ± 0.39) μg · mL^−1^ in the icariin group, (2.37 ± 0.74) μg · mL^−1^ in the 0.9% NaCl group, and (0.99 ± 0.24) μg · mL^−1^ in the healthy group, which indicates a significant decrease in IL-1β expression after icariin administration compared to 0.9% NaCl and recovery to near healthy levels (Fig. 4a). The ELISA results showed a decrease in IFN-γ expression in the icariin group, but there was no significant difference between the three groups (Fig. 4b). The ELISA did not detect tumor necrosis factor-alpha (TNF-α) expression.

Routine blood, immunoglobulin and biochemical tests of whole blood were examined. Routine blood indicators, immunoglobulins and routine blood biochemical indicators were not significantly different between the 0.9% NaCl group, icariin group and healthy group (Supplementary Tables 1–3).

## Discussion

Periodontitis may lead to the destruction of periodontal tissues, including periodontal ligament, alveolar bone, and cementum. The ultimate goal of periodontal treatment is the regeneration of supporting periodontal tissues. Large animals are superior to small animals for pre-clinical investigations of periodontal regeneration.^17^ There are many similar characteristics in the physiology and pathophysiology of periodontitis in humans and minipigs.^17,18^ Therefore, we generated a minipig model of periodontitis via the surgical creation of periodontal defects and a silk suture to generate periodontitis in a short time to determine whether icariin regenerated periodontal tissues.^19^

The candidate concentrations of icariin for periodontal tissue regeneration were investigated. Drug-stimulated periodontal tissue regeneration requires the recruitment and activation of local endogenous MSCs, such as PDLSCs, which differentiate into cementoblasts, periodontal ligament cells, and osteoblasts.^4^ Our previous study demonstrated that icariin promoted the proliferation and osteogenic differentiation potentials of hPDLSCs in inflammatory conditions at an optimal concentration of 0.1 μg · mL^−1^.^13^ Therefore, the present study used local injections of 0.1 μg · mL^−1^ icariin. Clinical assessment, CT scan and histopathological results demonstrated that local icariin injection significantly promoted the regeneration of periodontal tissues, including increased new alveolar bone formation and a more mature and thicker new cementum and typical structure of Sharpey’s fibres in a minipig model of periodontitis. These results suggest that icariin enhanced periodontal tissue regeneration via the promotion of endogenous MSC function, such as proliferation and directed differentiation. Several studies demonstrated that icariin promoted bone formation and inhibited bone resorption in in vivo investigations, which also supports our results that local icariin application enhanced alveolar bone formation in a minipig model of periodontitis.^14,20^

Another key issue in ideal periodontitis treatment is the alleviation of local inflammation and regulation of the local immune reaction. We investigated the effect of icariin on inflammation and the immune reaction. Local icariin injection improved the colour, shape and texture of gums on gross observation and reduced the number of local inflammatory cells in histopathological photomicrographs, which indicates an alleviation of local inflammation in our minipig model of periodontitis. Inflamed tissues, such as gums in periodontitis, produce high levels of pro-inflammatory factors, including IL-1β, TNF-ɑ and IFN-γ, and low levels of anti-inflammatory factors.^21^ Several studies demonstrated the presence of T regulatory cells (Tregs) in periodontal tissues, which play an important role in controlling periodontal infection and protecting against tissue destruction.^22,23^ The expression of IFN-γ was excessive because of the restrained function of Tregs, which was associated with severe destructive periodontal tissues. The excessive expression of IFN-γ activated macrophages to promote the secretion of inflammatory cytokines, such as IL-1β, TNF-α and prostaglandin E_2_ (PGE_2_).^24,25^ Macrophages and fibroblasts produce TNF-α during immune responses, which downregulates osteoblastogenesis and up-regulates osteoclastogenesis.^26,27^ IL-1β acts synergistically with TNF-α in inflammatory and immune responses.^28^ All of these cytokines are important in balancing the formation and resorption of alveolar bone.^21,22,29–31^

IL-1β, TNF-α and IFN-γ in GCF were examined to confirm the effects of icariin on inflammation and immunity. TNF-α was not detectable in our minipig model of periodontitis, but the ELISA demonstrated that local icariin significantly decreased IL-1β expression, which indicates that icariin regulated inflammatory and immune reactions in periodontitis. Our results demonstrated that icariin administration inhibited IFN-γ expression, but this inhibition was not significant. Our exploratory study did not establish the maintenance time or dose effect of icariin administration via simple local injection in periodontal tissues. Future studies will examine different application techniques of icariin, such as dose, period and release method, to obtain a more powerful understanding of its effects.

Previous studies found that icariin inhibited inflammatory responses, osteoclast differentiation and bone resorption via suppression of TNF-α and IL-1β synthesis, which are regulated by the mitogen-activated protein kinases/nuclear factor-kappa B (MAPKs/NF-κB) pathway.^20,32^ Icariin also exerted inhibitory effects on the TNF-α/IFN-γ-induced inflammatory response and reduced the expression of inflammatory mediators, such as IL-1β, via inhibition of the substance P and the p38 mitogen-activated protein kinase (p38-MAPK) signalling pathway.^25^ Foxp3 also plays a key role in the development and function of Treg cells, which are regulated by IL-1β, and deficiency promoted the inflammatory response.^22,33^ It has been reported that icariin promotes the expression of Foxp3, which suggests that icariin exerted its anti-inflammatory ability via regulation of Th17 and Treg cells.^34^ Taken together, these results indicated that icariin exhibits inflammatory and immune regulatory effects in a minipig model of periodontitis, and the MAPK signalling pathway and Foxp3 may play a role in this process in periodontitis. However, further investigations are required to clarify this issue.

In conclusion, the present study demonstrated that local icariin injection promoted periodontal tissue regeneration and exerted anti-inflammatory and immunomodulatory functions in a minipig model of periodontitis. These results support the potential application of icariin for periodontitis treatment in the future.

## Materials & methods

### Reagents

Icariin, with a purity of 98.5%, was obtained from the National Institute for the Control of Pharmaceutical and Biological Products (NICPBP; Beijing, China). Icariin powder was sealed and stored in the dark at 4 °C. Icariin was dissolved in phosphate-buffered saline (PBS; Invitrogen, CA, Carlsbad, USA), and the final concentration used was 0.1 μg · mL^−1^.

### Animals

Nine adult and healthy inbred female minipigs (12–18 months old, weighing 50–55 kg) were obtained from the Institute of Animal Science of Beijing Shichuang Century Minipig Breeding Base (Beijing, China). Animals were raised under conventional conditions with free access to water and standard mixed feed. This project was approved by the animal care and use committees of Capital Medical University School of Stomatology, Beijing, China (KQYY-201608-002).

### Establishment of a periodontitis model

Six minipigs were used to generate a periodontitis model, and a total of 12 periodontal defects were established. These minipigs were randomly divided into an experimental group and a control group (six periodontal defects in three minipigs in each group). The other three minipigs were used as the healthy control group and sacrificed without any treatment at the end of the study. The minipigs were anaesthetized with a combination of xylazine (0.6 mg · kg^−1^; Huamu, Changchun, China) and Zoletil 50 (10 mg · kg^−1^; Virbac, Carros, France). Animals were clinically assessed, and alveolar bone was removed using a surgical process to create experimental periodontal defects in the mesiobuccal region of the bilateral mandibular first molars. The created alveolar bone defect was 7 mm in width, 5 mm in depth, and 7 mm in length (Fig. 1a). A 4–0 silk ligament was sutured around the cervical portion of the bilateral mandibular first molars. The postoperative diet was routine and without antibiotics. Animals with experimental periodontitis were randomly assigned to two groups: injection of 0.9% NaCl (0.9% NaCl group) or injection of 0.1 μg · ml^−1^ icariin (icariin group). Minipigs with periodontitis were injected at three sites surrounding each periodontal defect four weeks after surgery: the distal side of the molar, the mesial side of the molar, and the middle of the molar. Each injection needle was inserted from the mucosa to the surface of the bone (supraperiosteal), and 0.9% NaCl or icariin was injected after significant resistance was encountered. Minipigs with periodontitis were injected with 0.9% NaCl or icariin every two weeks and sacrificed 12 weeks after injection.

### Clinical and radiological evaluations

Clinical assessments, including AL, GR, and PD, were recorded in all experimental teeth pre-operation (−4 weeks), pre-injection (0 weeks) and post-injection (12 weeks) using a Williams periodontal probe (Shanghai Kangqiao Dental Instruments Factory, Shanghai, China). Computed tomography (CT; Siemens, Erlangen, Germany) scans were performed at a scanning length of 0.625 mm at pre-injection (0 weeks) and post-injection (12 weeks) to examine bone regeneration.

### Local inflammatory factor assessments

GCF was collected from minipigs periodontal pockets using periopapers 12 weeks after injection. These samples were weighed and stored at −80 °C.ELISA (R&D Systems, Minneapolis, MN, USA) was used to examine the content of TNF-α, IL-1β, and IFN-γ in the GCF.

### Histological assessments of regenerated periodontal tissues

All animals were sacrificed 12 weeks after injection. Experimental samples were fixed in 4% formaldehyde and decalcified in buffered 50% formic acid. Experimental tissues were subsequently embedded in paraffin and stained with haematoxylin and eosin (HE). Buccal-lingual direction sections of experimental regions were created for histological analyses, and the thickness of tissue slices was 5 μm.

### Statistical analysis

Statistical analysis was performed using Student’s *t* test and one-way analysis of variance in Statistical Product and Service Solutions version 17.0 (SPSS 17.0, Chicago, IL, USA) statistical software. A *P* value < 0.05 was considered a statistically significant difference.

## Electronic supplementary material

supplementary table 1

supplementary table 2

supplementary table 3
