# Supplementary material for: Local icariin application enhanced periodontal tissue regeneration and relieved local inflammation in a minipig model of periodontitis
Source: Int J Oral Sci. 2018 Jun 12;10(2):19. doi: 10.1038/s41368-018-0020-3 (PMC5997630; doi:10.1038/s41368-018-0020-3)
Supplement: Supplementary file 1 — supplementary table 1 [file 41368_2018_20_MOESM1_ESM.docx]

**Supplementary table 1.The results of routine blood tests at the time point of post-injection**

| Project name | Healthy group | 0.9% NaCl group | Icariin group |
| --- | --- | --- | --- |
| RBC*(10^^12^/L) | 6.60±1.89 | 7.05±0.26 | 6.45±0.20 |
| MCV*(fl) | 54.70±3.17 | 55.43±3.52 | 54.00±2.09 |
| RDW-CV*(10^^9^/L) | 19.37±0.55 | 20.37±0.61 | 19.93±0.74 |
| RDW-SD*(%) | 43.97±4.63 | 49.27±6.64 | 47.87±3.09 |
| HCT*(%) | 36.07±1.31 | 39.17±3.67 | 34.83±0.76 |
| PLT*(10^^9^/L) | 432.33±87.52 | 415.00±99.54 | 407.67±101.32 |
| MPV*(fl) | 7.73±0.21 | 8.47±0.93 | 9.13±0.21 |
| WBC*(10^^9^/L) | 17.60±1.99 | 17.07±2.51 | 17.60±2.30 |
| HGB*(g/l) | 12.50±0.44 | 13.53±1.19 | 12.30±0.44 |
| MCH*(pg) | 18.97±0.90 | 19.17±1.08 | 19.07±0.38 |
| MCHC*(g/l) | 34.73±0.61 | 34.60±0.53 | 35.30±1.08 |
| LYM*(10^^9^/L) | 9.33±1.06 | 9.10±1.54 | 9.23±0.35 |
| GRAN*(10^^9^/L) | 6.73±0.85 | 6.60±1.21 | 6.83±1.80 |
| MONO*(10^^9^/L) | 1.47±0.21 | 1.37±0.21 | 1.53±0.15 |
| LYM%*(%) | 53.10±2.78 | 53.30±4.88 | 53.10±5.15 |
| GRA%*(%) | 38.37±1.86 | 38.80±4.33 | 38.53±5.03 |
| MON%*(%) | 8.53±1.10 | 7.90±0.62 | 8.37±0.15 |

*P>0.05, there was no significant difference among these three groups at the time point of post-injection.
