# Supplementary material for: Local icariin application enhanced periodontal tissue regeneration and relieved local inflammation in a minipig model of periodontitis
Source: Int J Oral Sci. 2018 Jun 12;10(2):19. doi: 10.1038/s41368-018-0020-3 (PMC5997630; doi:10.1038/s41368-018-0020-3)
Supplement: Supplementary file 2 — supplementary table 2 [file 41368_2018_20_MOESM2_ESM.docx]

**Supplementary table 2.The results of biochemical tests at the time point of post-injection**

| Project name | Healthy group | 0.9% NaCl group | Icariin group |
| --- | --- | --- | --- |
| TP*(g/l) | 70.73±3.33 | 71.07±0.85 | 71.8±1.80 |
| ALB*(g/l) | 40.27±0.87 | 40.47±0.61 | 40.37±0.85 |
| GLB*(g/l) | 30.47±2.54 | 30.60±0.62 | 31.43±1.65 |
| A/G* | 1.32±0.13 | 1.33±0.02 | 1.29±0.08 |
| ALT*(U/L) | 65.00±11.53 | 65.67±9.71 | 64.33±8.14 |
| AST*(U/L) | 74.33±11.15 | 70.33±3.51 | 70.67±8.14 |
| ALP*(U/L) | 62.00±5.29 | 69.00±9.64 | 68.00±11.53 |
| K+*(mmol/L) | 3.51±0.53 | 3.43±0.28 | 3.34±0.14 |
| Na+*(mmol/L) | 142.33±5.03 | 144.33±6.03 | 136.67±2.31 |
| Cl-*(mmol/L) | 100.67±4.73 | 101.67±6.43 | 98.33±1.53 |
| CO2*(mmol/L) | 30.57±0.83 | 27.90±1.65 | 28.67±2.73 |
| Ca^2+^*(mmol/L) | 2.39±0.32 | 2.41±0.15 | 2.29±0.09 |
| P*(mmol/L) | 2.34±0.53 | 2.58±0.19 | 2.26±0.16 |
| GLU*(mmol/L) | 5.33±0.53 | 4.99±0.30 | 4.63±0.49 |
| CREA*(umol/L) | 94.93±9.65 | 91.90±4.25 | 92.57±7.11 |
| BUN*(U/L) | 4.33±0.78 | 4.60±0.70 | 4.23±0.68 |
| LDH*(U/L) | 580.67±88.94 | 616.67±121.89 | 492.33±48.17 |
| AMY*(U/L) | 1025.67±66.76 | 973.66±144.56 | 1064.33±80.99 |

*P>0.05, there was no significant difference among these three groups at the time point of post-injection.
