# Supplementary material for: Local icariin application enhanced periodontal tissue regeneration and relieved local inflammation in a minipig model of periodontitis
Source: Int J Oral Sci. 2018 Jun 12;10(2):19. doi: 10.1038/s41368-018-0020-3 (PMC5997630; doi:10.1038/s41368-018-0020-3)
Supplement: Supplementary file 3 — supplementary table 3 [file 41368_2018_20_MOESM3_ESM.docx]

**Supplementary table 3. The results of immunoglobulin tests at the time point of post-injection**

| Project name | Healthy group | 0.9% NaCl group | Icariin group |
| --- | --- | --- | --- |
| IgA*(mg/ml) | 7.41±0.44 | 8.25±1.41 | 8.09±1.05 |
| IgE*(ng/ml) | 8.54±0.54 | 8.75±0.40 | 8.61±0.32 |
| IgM*(ug/ml) | 0.30±0.02 | 0.32±0.03 | 0.35±0.06 |
| IgG*(ug/ml) | 0.46±0.08 | 0.53±0.07 | 0.44±0.09 |

*P>0.05, there was no significant difference among these three groups at the time point of post-injection.
